# Skin disorders in diabetes mellitus: an epidemiology and physiopathology review

**DOI:** 10.1186/s13098-016-0176-y

**Published:** 2016-08-30

**Authors:** Geisa Maria Campos de Macedo, Samanta Nunes, Tania Barreto

**Affiliations:** 1 Endocrine and Diabetes Department, Agamenon Magalhães Hospital, Estrada do Arraial, 2723, Casa Amarela, Recife, PE 52070-230 Brazil; 2 Brazilian Society of Dermatology São Paulo, São Paulo, 05423 010 Brazil; 3 Diabetes Division, Sanofi, São Paulo, São Paulo 05693-000 Brazil

**Keywords:** Diabetes mellitus, Skin disorders, Non-pharmacological treatment

## Abstract

Skin disorders, usually neglected and frequently underdiagnosed among diabetic patients, are common complications and encounter a broad spectrum of disorders in both type 1 and type 2 diabetes mellitus (DM)—e.g. cutaneous infection, dry skin, pruritus. Skin disorders are highly associated with increased risk of important outcomes, such as skin lesions, ulcerations and diabetic foot, which can lead to major complications and revolve around multifactorial factors besides hyperglycemia and advanced glycation end products. Although diabetic’s skin disorders are consistent in the literature, there is limited data regarding early-stage skin disorders in DM patients. Disease control, early-stage treatment (e.g. skin hydration, orthotic devices) and awareness can reduce morbidity of DM patients. Thus, better understanding of the burden of skin disorders in DM patients may raise awareness on prevention and management. Therefore, the aim of this study is to perform a literature review to evaluate the main clinical characteristics and complications of skin disorders in diabetic’s patients. Additionally, physiopathology early-stage skin disorders and dermocosmetic management were also reviewed.

## Background

Skin disorders, usually neglected and frequently underdiagnosed among diabetic patients, are common complications and encounter a broad spectrum of disorders in both type 1 and type 2 diabetes mellitus (DM)—e.g. cutaneous infection, dry skin, pruritus—that can lead to major complications and are highly associated with hyperglycemia and advanced glycation end products (AGEs) [1].

Although diabetic’s skin disorders are consistent in the literature, there is limited data regarding early-stage skin disorders in DM patients, especially focusing on non-injured skin [2]. Better understanding of the burden of skin disorders in diabetes patients may raise awareness on prevention and management. Therefore, the aim of this study is to perform a literature review to evaluate the main clinical characteristics and complications of skin disorders in diabetic’s patients. Additionally, management of skin disorders was also reviewed.

## Skin disorders in diabetes and epidemiology

Diabetes mellitus (DM) represents a high prevalent disease with high morbidity and mortality. In 2014, there were 387 million diagnosed cases of diabetes and 4.9 million deaths worldwide. Additionally, about 77 % of people with diabetes live in less developed regions [3]. Although prevalence of diabetes morbidity is high, specific data on complications related to skin disorders are limited. Several epidemiological studies evaluating occurrence of skin disorders on type 1 and type 2 DM were performed worldwide, with pattern of skin disorders varying according to DM type and region where the study was conducted.

Overall prevalence of skin disorder in both type 1 and 2 DM varied from 51.1 to 97 % in different regions worldwide. The high prevalence of dermatological disorder among DM patients described in literature endorses the clinical importance and high impact of this complication.

Although study design and eligibility criteria of the included patients varied slightly among reported studies, most frequent disorder reported in diabetic patients, regardless of DM type, was infection—occurring in at least 20.6 % of diagnosed patients. Moreover, fungal infections were more prevalent than bacterial or viral infections [4–8], and interdigital spaces, genitalia and skin folds were the most frequent site of infection [4].

In a single center epidemiologic study conducted in Iran, infection was also the most common lesion reported by patients—in this study, the most common noninfectious manifestation was pruritus [9]. Similarly, Sasmaz et al. showed that most common skin conditions in DM patients are infections (31.7 %), non-candidal intertrigo (20.5 %), eczemas (15.2 %), psoriasis (11.2 %), diabetic dermopathy (11.2 %), and prurigo (9.9 %) [5].

A study conducted in Brazil evaluated 403 patients with type 1 (n = 125) and type 2 (n = 278) DM assisted in the outpatient clinic from Ribeirão Preto Hospital in 2000. The study demonstrated that 81 % of patients had at least one dermatologic lesion, with a mean of 3.7 lesions per patient, being dermatophytosis the most common lesion. Of all dermatophytosis, 42.6 % were onychomycoses (n = 172) and 29.2 % were tinea pedis (n = 118). Skin lesions occurring in more than 10 % of the patients were actinic degeneration (62 %), skin xerosis (20.8 %), benign skin tumor (23.5 %), candidiasis (12.9 %) and scar (12.6 %) [6].

Another study in Brazil, also conducted in the outpatient clinic from Ribeirão Preto Hospital from 2003 to 2004, evaluated 500 DM patients. The study demonstrated that 97 % of all patients had at least one skin lesion—the highest skin disorder rate in this review—being tinea pedis (35 %), candidiasis of the skin/nail (26 %), pigmentation disorders (22 %), xerosis (22 %) and tinea unguium (22 %) the most commonly reported dermatological diagnoses among DM patients [10].

A smaller study in Brazil conducted in Canoas with 55 patients also demonstrated a high prevalence of skin disorders among DM patients (89.1 %), comprising of yellow nails (52.7 %), candidiasis (52.7 %), dermatophytosis (50.9 %), nail dystrophy (45.5 %) and *Staphylococcus* infections (38.2 %) [11].

Galdeano et al. evaluated 125 patients with type 1 and 2 DM in a single center in Argentina. The study showed a high prevalence of skin disorders: 90.4 %. Skin disorders occurring in more than 10 % of the patients included xeroderma (69 %), dermatophytosis (52 %), onychomycosis. (49 %), tineapedis (39 %), peripheral hypotrichia (39 %), diabetic dermopathy (35 %) skin thickening syndrome (25 %), diabetic foot (24 %), candidiasis (17 %), fibroids pendulums (11 %), intertrigo (10 %), and inner eyebrow separation (10 %) [12].

In Egypt, Sanad et al. evaluated 100 patients diagnosed with type 1 (n = 23) and type 2 (n = 77) DM, with at least one skin lesion, in a single center cohort study. The mean time of diagnosis was 10.57 ± 7.63 years. In this study, the most common skin disorders were cutaneous infections (40 %), followed by pruritus (11 %), local reactions at the site of insulin injection (8 %), vitiligo (8 %), diabetic dermopathy (7 %), periungual telangiectasia (6 %), and xanthelasma (5 %). Xerosis was reported in only 3 % of patients. Cutaneous infections included fungal (22 %), bacterial (16 %), and viral (2 %) infections. Tinea pedis was the most common fungal infection (12 %), whereas boils were the most common bacterial infection (5 %). Among viral infections, one patient had herpes simplex and another had herpes zoster [7].

Differences between patterns of lesions remain unclear among types of diabetes. A total of five studies evaluated skin disorders in both types 1 and 2DM. Chattergee et al. showed higher prevalence of skin disorder in type 2 DM (75.6 vs 41 %). In this same study, the most common skin disorders on type 1 DM were diabetic xerosis, infections and diabetic hand. Differently, most frequent disorders presented in patients with type 2 DM were infections, xerosis, hair loss below the knees and diabetic dermopathy [13].

One case–control study evaluating type 1 DM in young patients showed that the most prevalent skin disorder was xerosis, occurring in 22.2 % of patients compared to 3 % in the control group (p < 0.01) [14]. Another study evaluating type1 DM in young patients (n = 500), conducted in South Asia, demonstrated that the most common disorders related to the disease were limited joint complication (16.8 %), xerosis (15.8 %) and scleroderma-like skin changes (10 %). The author also reported complications related to disease treatment, which included lipohypertrophy (41 %), postinflammatory hyperpigmentation (3 %) and lipoatrophy (0.6 %) [15].

Additionally, Farshchian et al. also reported differences on patterns of infection according to the types of diabetes. In the type 1 DM, the most frequent cutaneous infections were viral warts, while pyodermas were the most frequent cutaneous infections in type 2 DM patients [9]. Although prevalence of skin disorders appears to be higher in type 2 DM, these disorders should be monitored in early stage regardless of type of diabetes and manifestation.

Overall, cutaneous infection and xerosis showed to be highly prevalent and important skin disorders in several studies, regardless DM type. Among cutaneous infections, fungal etiology appears to be the most common and those with bacterial origin are the less frequent [4–8]. Other outcomes such as xeroderma, reactions related to the treatment, eczema, pruritus, xanthelasma, and diabetic dermopathy were also reported and should be monitored too.

Although prior studies showed increased risk of infection in DM patients [16], little evidence is found in the literature to support increased risk for cutaneous infectious diseases [9]. In general, skin disorders are highly associated to poorly controlled DM patients. A good glycemic control may reduce the incidence and severity of cutaneous disorders with or without known pathogenesis [17]. Nonspecific skin disorders that occur in DM patients can increase the likelihood of exposure to infectious organisms and contact with allergens, resulting in chronic and recurrent infections and eczemas, respectively [17]. However, further studies are required, since all available data are still not concordant. Additionally, Farshician et al. did not find a significant relationship between the diabetic disease control and the prevalence of cutaneous infection [9]. Moreover, most cross-sectional studies assessed in this review do not allow causal explanations. Thus, further investigation is required to better understand the risk of cutaneous infection.

Xerosis was reported in several studies and rates showed high heterogeneity. Goyal et al. showed a high prevalence of xerosis (44 %) in a single center observational study mainly related to weather and dry climatic conditions [8]. According to Pavlovic, skin dryness is one of the earliest and most common manifestations of type 1 DM [14]. Clinical observations are supported by a reduced hydration state of the stratum corneum and decreased sebaceous gland activity in DM patients, without any impairment of the stratum corneum barrier function. Even in the absence of clinically apparent xerosis, patients with diabetes have an impaired desquamation process. Furthermore, occurrence of xerosis may be affected not only by the type of diabetes but also to regional changes in climate and humidity [7].

## Major pathways on skin disorders in DM

Skin disorders in DM patients are highly correlated with glycemic control. As an example, Foos et al. conducted a study with 403 DM patients in Brazil and evaluated their skin disorders and glycaemia control. Thus, the study demonstrated that 94 % of patients with inadequate glycaemia control had some skin disorder; on the other hand, only 60 % of DM patients with adequate glycaemia control had some skin disorder [6].

DM affects the skin through several mechanisms, being hyperglycemia per se and AGEs the most well-described. Reaching pathological high levels of glycaemia strongly affects skin homeostasis by inhibiting keratinocyte proliferation and migration, protein biosynthesis, inducing endothelial cell apoptosis, decreasing nitric oxide synthesis and impairing phagocytosis and chemotaxis from several cells [18, 19]. Besides hyperglycemia induce direct damage, high glucose levels also induce AGE formation. AGEs are formed from glycation of proteins, lipids and nucleic acids [18, 19] that act in several pathways, inducing reactive oxygen species (ROS) formation, impairing ROS clearance, as well as intra and extracellular proteins function, and inducing pro inflammatory cytokine through nuclear factor κβ (NF-κβ) pathway [20].

Indeed, AGE biochemical interactions are one of the major pathways involved in DM complications, including skin disorders [21]. AGE alters collagen properties, decreasing flexibility and solubility and increasing its rigidity [22]. Also, AGEs participate in the development of fibrosis in DM [23], in skin aging [20] and even in diabetes-related immunosuppression [24]. Diabetes-related immunosuppression affects skin wounding, mainly by leukocyte impaired function and misbalanced/malfunction of growth factors [25].

In addition to the previously mentioned pathways, high glucose levels also impair the normal functioning of keratinocytes in vitro, decreasing its proliferation and differentiation [26, 27]. Moreover, keratinocytes studies are more commonly conducted in animal models, with scarce data on diabetic human skin [2]. Regarding epidermal thickness alterations, Bertheim et al. demonstrated that diabetic patients with severe joint mobility on the hands had an increased epidermal thickness, with abnormal hyaluronan distribution on skin layers [28], while Zakharov et al. demonstrated that well-controlled patients with type 1 DM did not presented alteration on the epidermal thickness [29]. This data reinforces that skin disorders in DM patients are strongly related to glycaemia control.

Table 1 summary of studies reporting skin disorders prevalence in DM 1 and DM 2 patients, with a subjects description (number of subjects in the study, type of DM evaluated, country/region where the study was conducted, subjects mean age), prevalence of skin condition and the most common skin condition in each study.

**Table 1:** Summary of studies reporting skin disorders prevalence in DM 1 and DM 2 patients, with a subjects description (number of subjects in the study, type of DM evaluated, country/region where the study was conducted, subjects mean age), prevalence of skin condition and the most common skin condition in each study

Author	Sample size	DM type	Country/region	Age (years)	Overall prevalence (%)	Overall most frequent skin disorder among all disorders (%)
Sasmaz et al. [5]	151	Type 2	India	54 ± 17	85.4	Cutaneous infection (20.6 %)
Chatterjee, et al. [13]	680	Types 1 and 2	India	46.3 ± 6.7	73.9	Cutaneous infection (40.9 %)
Farshchian et al. [9]	155	Types 1 and 2	Iran	21.8 ± 4.9 and 57.2 ± 9.7, type 1 and 2, respectively	71	Cutaneous infection
Foss et al. [6]	403	Types 1 and 2	Brazil	19.9 ± 2.3 and 63.1 ± 3.4 type 1 and 2, respectively	81	Cutaneous infection (82.6 %)
Wambier et al. [10]	500	Types 1 and 2	Brazil	45.5 ± 20	97	Cutaneous infection (i.e.Tinea pedis) (35 %)
da Silva et al. [11]	55	Type 2	Brazil	56.3 ± 13.4	89.1	Yellow nails (52.7 %) and candidiasis (52.7 %)
Galdeano et al. [12]	125	Types 1 and 2	Argentina	58.9 ± 15.43	90.4	Xeroderma (69 %)
Pavlovic et al. [14]	212	Type 1	Servia	12.5 ± 3.7	68	Xerosis (22.2 %)
Romano et al. [4]	457	Types 1 and 2	Italy	61.5 ± 11.3	60	Cutaneous infection
Sanad et al. [7]	100	Types 1 and 2	Egypt	51.42 ± 14.66	–	Cutaneous infection (40 %)
Sawatkar et a [15]l	500	Type 1	South Asia	16.9 ± 6.9	67.8	Limited joint mobility (16.8 %)
Goyal et al. [8]	100	Types 1 and 2	India	57.44 ± 10.37	–	Xerosis (44 %)
Ragunatha et al. [17]	500	Types 1 and 2	India	55.24 ± 11.24	51.1	Cutaneous infection

A summary of skin evaluation studies performed in humans is described in Table 2 [28–33].

**Table 2:** Summary of findings on skin alterations in DM patients

Parameter	Effect in DM patients	Reference
Hydration	Decreased	Sakai et al. [30]
	No alteration	Seirafi et al. [31]
Trans-epidermal loss	Not altered	Sakai et al. [30]
		Seirafi et al. [31]
Filaggrin	Alteration	Thyssen et al. [32]
Other possible signs of barrier defect	Increased inflammatory infiltration	Tellechea et al. [33]
Epidermal thickness	No alteration	Zakharov et al. [29]
	Thicker epidermis	Bertheim et al. [28]

Adapted from Quondamatteo [2]

Cutaneous manifestations in DM patients are mainly classified into four categories in order to support management of the outcomes, especially due to innumerous potential causes:

1. Skin lesions with strong to weak association with diabetes (necrobiosis lipoidica, diabetic dermopathy, diabetic bullae, yellow skin, eruptive xanthomas, perforating disorders, acanthosis nigricans, oral leucoplakia, lichen planus).
2. Infections (bacterial, fungal).
3. Cutaneous manifestations of diabetic complications (microangiopathy, macroangiopathy, neuropathy).
4. Skin reactions to diabetic treatment (sulphonylureas or insulin) [4].

Considering cutaneous manifestations on DM, some skin conditions are more likely to affect type 1 or 2 DM disproportionally. Although with high inter-study variability, skin manifestations demonstrating association with DM type are described in Table 3 [34].

**Table 3:** Most frequent skin disorders among type 1 and type 2 DM

Type 1 diabetes mellitus	Type 2 diabetes mellitus
Necrobiosis lipoidica diabeticorum	Generalized granuloma annulare
Diabetic bullae	Scleredema diabeticorum
Vitiligo vulgaris	Diabetic dermopathy
Periungual telangiaecstasia	Acathosis nigricans
–	Acrochordons
–	Psoriasis

Adapted from Murphy-Chutorian et al. [32]

## DM skin disorders progression and potential outcomes

**Fig. 1:**
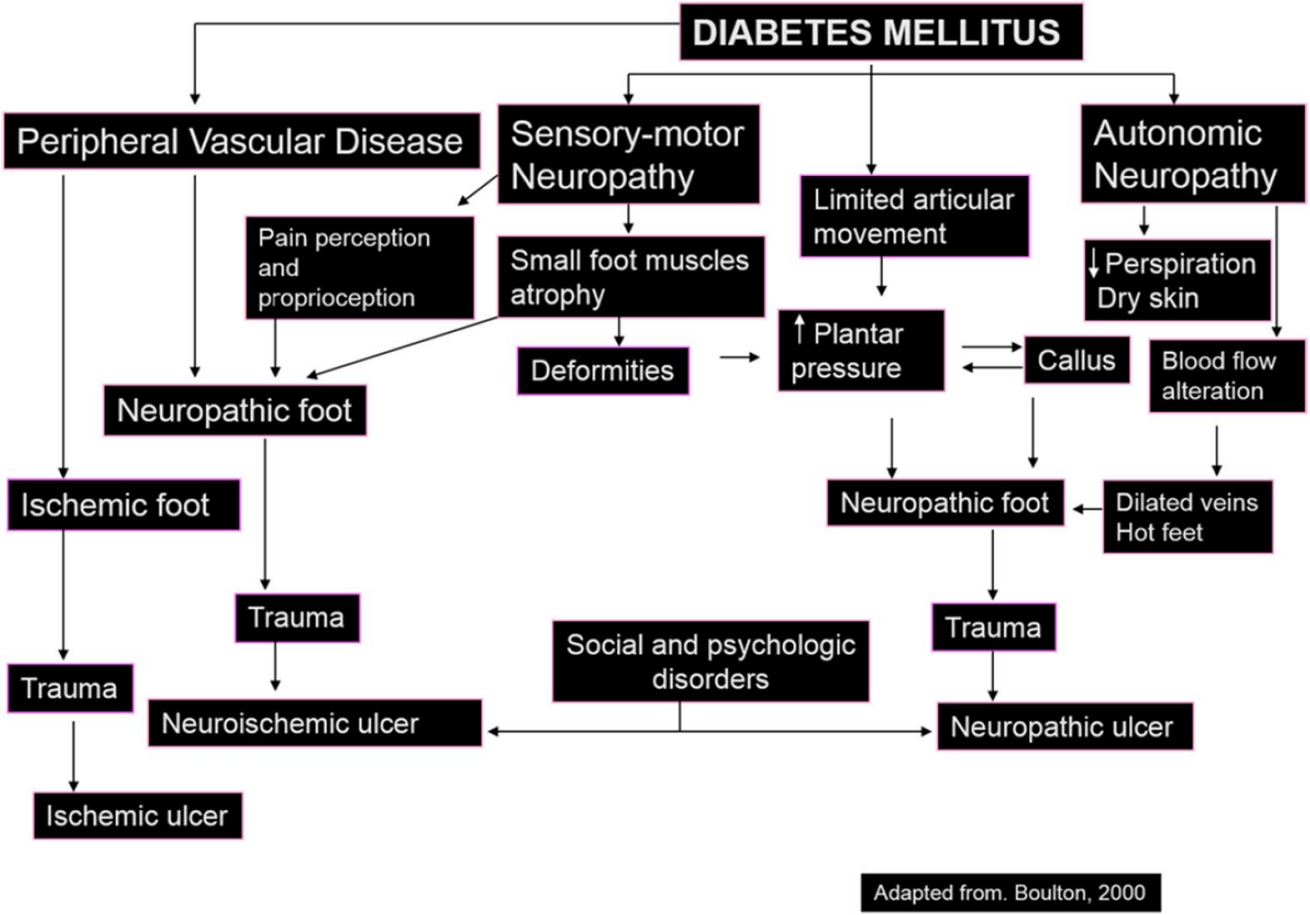
Neuropathic foot ulcer pathophysiology. Neuropathic foot ulcer physiopathology pathways, with autonomic, motor and sensory neuropathies leading to foot neuropathy. Adapted from Boulton [37]

An important variability on severity and potential outcomes is observed among skin disorders in DM. Early-stage skin disorders in DM, such as xerosis, callus and fissures, are usually neglected and frequently underdiagnosed [1]. Lack of diagnosis and treatment on early-stage skin disorders can lead to clinical worsening, and progression to foot neuropathy, ulcers and even amputation [35]. DM-induced neuropathy can reach sensory, motor and autonomic pathways, leading to different dermatologic conditions (Fig. 1).

- Sensory neuropathy: insensibility and decreased temperature sensation, affecting the sensibility on lesions [36, 37].
- Motor neuropathy: causes toe and gait deformity, leading to foot deformity and increased plantar pressure [38, 39].
- Autonomic neuropathy: leads to anhidrosis and vasodilation, causing dry skin, skin tears and fissures [40], also losing viscoelasticity [41].

All the neuropathies, alone or simultaneously, can lead to neuropathic foot ulcer, the most severe cutaneous lesion, in consequence of the poor healing potential of DM skin [42] that frequently become infected, thus leading to amputation [43, 44].

Complications prevention by treating chronic hyperglycemia and early-stage symptoms (e.g. skin hydration, orthotic devices, patient education) is highly acclaimed by medical society, once the diabetic foot brings severe economic, personal, social, and medical impact [35, 45]. Furthermore, xerosis, callus and foot deformity are the early-stage complications target to avoid the development of diabetic foot. Besides, a continuous patient education is also required to ensure that patients take proper care of the skin and foot.

Although several clinical outcomes related to DM, many cellular impairments are also related to hyperglycemia and hyperinsulinemia. As an example, keratinocytes, which is the predominant cell type in the epidermis, are downregulated in hyperglycemia conditions, with impairment on its proliferation [26]. In addition, a study performed in animal model demonstrated an imbalance on stratum corneum composition (lower triglycerides and higher ceramides, cholesterol and fatty acids levels), when compared to control. Furthermore, another study showed an increased number of corneocyte layers in the stratum corneum and decreased basal cell proliferation and epidermal DNA content—important in epidermal differentiation—suggesting slower stratum corneum and epidermal turnover [46]. Moreover, larger corneocyte surface area is observed in DM patients, when compared to control [47].

The dermis also suffers morphological and biochemical alteration in DM patients due to the synthesis and/or degradation of intracellular matrix and microarchitectural arrangement. Moczar et al. [48] demonstrated that skin biopsies of DM patients had ultrastructural modifications on fibroblast, collagen and elastic fibers in the dermis, with fragmented or absent elastic fibers under the epithelial basal laminae. Additionally, DM patients’ dermis presented collagenase and elastase alterations, endorsing the important matrix macromolecules alterations on DM patient’s dermis

As a result of all those changes, lower plasticity of the epidermis is seen in DM patients when compared to controls [49], without any alteration in transepidermal water loss (TEWL) [30, 31]. Furthermore, skin surface lipids appeared to be reduced in DM patients [30, 31]; also, another study demonstrated decreased skin elasticity in the forearm and forehead. Some of the functional properties of the stratum corneum in DM patients vary according to acute uncontrolled glycaemia (fasting plasma glucose > 110 mg/dL) and disease control (HbA1c > 5.8 %). In DM patients with fasting plasma glucose higher than 110 mg/dL, skin surface hydration was lower than in DM patients with fasting plasma glucose lower than 110 mg/dL, with no alteration on TEWL. On the other hand, patients with HbA1c levels higher than 5.8 % have slightly lower TEWL on forearm, with no alteration on skin surface hydration, when compared to patients with HbA1c < 5.8 % [30].

In conclusion, dermocosmetic strategy is an option to treat the biophysical alterations on DM skin, with efficacy on improving skin hydration, decreasing xerosis and scaling among other factors [50].

## Skin hydration: avoiding the progression

Dermocosmetic management through skin hydration and control of xerosis and callus symptoms are essential to avoid progression of skin lesions on DM patients [51]. Regarding dermocosmetic approach, the most commonly used active ingredient is urea. In 2003, Schölermann et al. published the results of two studies with 10 % urea cream in dry skin treatment. In the first study, 603 patients with dry or extremely dry skin, of which 179 were DM patients, were treated with 10 % urea cream for 14 days, presenting a decrease on dryness, callosities and scaling compared to baseline. In the second study, 30 patients with diabetes and/or xerosis were treated in one foot (the other foot was used as control) with 10 % urea cream for 10 days, resulting in decreased callosities, dryness and scaling [52].

Federici et al. conducted a randomized controlled clinical trial with 40 type 2 DM patients who were allocated to receive urea 5 %, arginine and carnosine versus a glycerol-based emollient for 28 days. Patients treated with urea 5 %, arginine and carnosine presented an 89 % reduction on dryness according to DASI scale when compared to control emollient [53]. Another study conducted with 54 type 1 and 2 DM patients treated with 10 % glycerin, 5 % urea, 1 % lactic acid (moisturizing agents) and 8 % paraffin (occlusive agent) in an emulsion base versus placebo (emulsion base with none of the active ingredients) for 4 weeks also demonstrated a decrease of dryness and fissures and increased skin hydration on active-treated group [54].

Similar studies were performed with 10 % urea and 4 % lactic acid [55]; 10 % urea, *Oenothera biennis* oil, *Centella asiatica* extract, α-hydroxy acid, allantoin, and panthenol formulation [56]; 5 % urea with 0.2 % hydroxyethylpiperazine ethane sulfonic acid [57]. Polaskova et al. performed a study comparing the 6 urea-containing different formulations for diabetic foot, evaluating TEWL, and demonstrated that urea 6 % formulations maintained or restored adequate barrier function, while urea 4 % formulations presented longer action period [58].

Recently, Nunes et al. published a study performed in 87 Brazilian DM patients evaluating efficacy and safety of cream (n = 43) or lotion (n = 44) Hidrastar formulations, containing urea, amino acids, linoleic acid, linolenic acid and SH-oligopeptide-2. After 28 days of application twice a day in both feet, 100 % of the patients using Hidrastar cream formulation reported increase in skin hydration, softness, improved texture and overall appearance. In the group using Hidrastar lotion formulation, 79.5 % of patients reported increased hydration, 81.8 % increase in softness, 81.8 % improvement in texture and 86.4 % improvement in the overall appearance of the skin of the feet [59].

## Conclusion

In conclusion, our review confirmed high prevalence of skin disorder in DM patients, showing that careful dermatological examination and outpatient follow-up of DM patients is required to provide them adequate skin management, thus reducing morbidity and complications related to skin. Additionally, in early-stage disorders, such as xerosis and callus, detection and management appeared to be important in reducing complications related to DM. This review summarizes the main articles in skin disorders and diabetes, with some limitations as the absence of statistical summary, from a meta-analysis. Further observational studies are required to clarify differences between types of diabetes and association between disease and risk of occurrence.

## Abbreviations

AGE: advanced glycation end-products
DASI: dryness area severity index
DM: diabetes mellitus
DM1: diabetes mellitus type 1
DM2: diabetes mellitus type 2
NF-κβ: nuclear factor κβ
ROS: reactive oxygen species
TEWL: transepidermal water loss
